# Pathogenic genomic alterations in Chinese pancreatic cancer patients and their therapeutical implications

**DOI:** 10.1002/cam4.5871

**Published:** 2023-03-31

**Authors:** Zhiming Zhao, Xiaomo Li, Fei Wang, Yong Xu, Si Liu, Quanli Han, Zhiying Yang, Weiwei Huang, Zhuzeng Yin, Qu Liu, Haidong Tan, Tonghui Ma, Shuang Si, Jia Huang, Hongling Yuan, Wei Li, Rong Liu

**Affiliations:** ^1^ Faculty of Hepatopancreatobiliary Surgery The First Medical Center of Chinese People's Liberation Army (PLA) General Hospital Beijing China; ^2^ Hangzhou Jichenjunchuang Medical Laboratory, Co. Ltd Hangzhou China; ^3^ Department of Medical Oncology The First Medical Center of Chinese People's Liberation Army (PLA) General Hospital Beijing China; ^4^ Department of Hepatobiliary Surgery China‐Japan Friendship Hospital Beijing China

**Keywords:** cancer risk, genetic alterations, next‐generation sequencing, pancreatic cancer, precision therapy

## Abstract

**Background:**

Approximately 90% of pancreatic ductal adenocarcinoma (PDAC) cases are driven by the untargetable non‐G12C *KRAS* mutations, and only a small subset of patients are eligible for FDA‐approved precision therapies. The practice of precision therapy in pancreatic cancer was limited by the paucity of targetable genetic alterations, especially in the Asian population.

**Methods:**

To explore therapeutic targets in 499 Chinese PDAC patients, a deep sequencing panel (OncoPanscan™, Genetron health) was used to characterize somatic alterations including point mutations, indels, copy number alterations, gene fusions as well as pathogenic germline variants.

**Results:**

We performed genomic profiling in 499 Chinese PDAC patients, which revealed somatic driver mutations in *KRAS*, *TP53*, *CDKN2A*, *SMAD4*, *ARID1A*, *RNF43*, and pathogenic germline variants (PGVs) in cancer predisposition genes including *BRCA2*, *PALB2*, and *ATM*. Overall, 20.4% of patients had targetable genomic alterations. About 8.4% of patients carried inactivating germline and somatic variants in *BRCA1/2* and *PALB2*, which were susceptible to platinum and PARP inhibitors therapy. Patients with *KRAS* wild‐type disease and early‐onset pancreatic cancer (EOPC) harbored actionable mutations including *BRAF*, *EGFR*, *ERBB2*, and *MAP2K1/2*. Compared to PGV‐negative patients, PGV‐positive patients were younger and more likely to have a family history of cancer. Furthermore, PGVs in *PALB2*, *BRCA2*, and *ATM* were associated with high PDAC risk in the Chinese population.

**Conclusions:**

Our results demonstrated that a genetic screen of actionable genomic variants could facilitate precision therapy and cancer risk reduction in pancreatic cancer patients of Asian ethnicity.

## Graphical abstract

Genomic profiling in 499 Chinese PDAC patients revealed somatic driver mutations in *KRAS*, *TP53*, *CDKN2A*, *SMAD4*, *ARID1A*, *RNF43*, and pathogenic germline variants (PGVs) in cancer predisposition genes including *BRCA2*, *PALB2*, and *ATM*. Overall, 20.4% of patients had targetable genomic alterations. Patients with KRAS wild‐type disease and early‐onset pancreatic cancer (EOPC) harbored actionable mutations including *BRAF*, *EGFR*, *ERBB2*, and *MAP2K1/2*.PGVs in *PALB2*, *BRCA2*, and *ATM* were associated with high PDAC risk in the Chinese population. Our results demonstrated that a genetic screen of actionable genomic variants could facilitate precision therapy and cancer risk reduction in pancreatic cancer patients of Asian ethnicity.

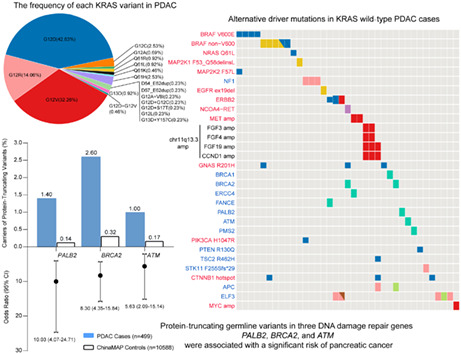

## 1 INTRODUCTION

Pancreatic cancer, with 90% cases being pancreatic ductal adenocarcinoma (PDAC), is a devastating disease with an overall 5‐year survival rate of <10%.^1^ Although precision medicine has shifted the treatment paradigm of many cancer types, its application was limited in PDAC. For instance, PDAC patients with *NTRK* fusions and high microsatellite instability (MSI‐H) or DNA mismatch repair deficiency (dMMR) are eligible for the treatment of NTRK inhibitors and PD‐1 antibody pembrolizumab, respectively.^2^, ^3^, ^4^ However, the prevalence of these biomarkers in PDAC patients was less than 2%.^2^, ^4^ Sotorasib and adagrasib, two KRAS G12C inhibitors with proven anti‐tumor activity in lung cancer, are under clinical investigation in PDAC (NCT03600883, NCT05251038, NCT03785249).^5^, ^6^ Nevertheless, the prevalence of *KRAS* G12C in PDAC is low (1%–3%).^4^ Therefore, there is an urgent need to identify new therapeutic targets for PDAC.^1^, ^4^

Approximately 10% of PDAC patients harbored pathogenic germline variants (PGVs) in cancer predisposition genes, many of which are targetable.^1^, ^2^, ^4^ Results of the phase 3 POLO trial showed that pancreatic cancer patients with *BRCA1*/*2* PGVs receiving PARP inhibitor (PARPi) olaparib had longer progression‐free survival (PFS) than placebo, although there was no difference in overall survival (OS) between the olaparib and placebo groups.^7^ Based on this data, both FDA and the NCCN guidelines endorsed olaparib as a second‐line therapy for this specific patient population.^2^ Additionally, pancreatic patients with Lynch Syndrome, a genetic disease mediated by PGVs in MMR genes (*MLH1*, *MSH2*, *MSH6*, *PMS2*), could benefit from immunotherapy.^2^ For cancer risk reduction and treatment purposes, the NCCN guidelines recommended universal germline testing for all pancreatic cancer patients.^2^ Furthermore, the NCCN guidelines also recommended somatic testing of *KRAS*, *BRAF*, *HER2*, *PALB2*, MMR deficiency, and oncogenic gene fusions for pancreatic cancer patients with advanced or metastatic diseases.^2^

The optimal clinical trial design for precision medicine requires the knowledge of actionable genomic variants in cancer patients of different ethnicities. Historically, genomic alteration data for PDAC were largely from patients of European origin, and there was a paucity of therapeutically relevant genomic variant data for Asian patients.^8^, ^9^, ^10^, ^11^ To address this issue, we set out to profile pathogenic germline and somatic genomic alterations in a large cohort of Chinese PDAC patients (*n* = 499) and drafted a molecular roadmap for the practice of precision medicine in PDAC.

## 2 MATERIALS AND METHODS

### 2.1 Patients

A total of 499 sporadic PDAC patients diagnosed between January 2019 and December 2021 were retrospectively studied. Both blood samples and tumor tissues were collected for germline/somatic testing. Demographic data of patients are listed in Table S1. The study protocol was approved by the Ethical Committee of the Chinese People's Liberation Army (PLA) General Hospital and the participants gave written informed consent before sample collection.

### 2.2 DNA sequencing

Total genomic and somatic DNA was extracted from 5 mL peripheral blood lymphocytes using a QIAamp DNA Mini Kit (QIAGEN) and from formalin‐fixed paraffin‐embedded (FFPE) tumor tissue using a GeneRead DNA FFPE Kit (QIAGEN), respectively. DNA samples were quantified with a Nanodrop 2000 spectrophotometer (Thermo Fisher Scientific) and qualified through Agilent 4200 TapeStation. DNA extracts were sheared into 150–200‐bp fragments using the S220 Focused‐ultrasonicator (Covaris). Libraries were prepared with the KAPA Hyper Prep Kit (KAPA Biosystems). Hybridization capture‐based targeted next‐generation sequencing (NGS) was processed on the Illumina NovoSeq 6000 platform. An 831‐gene panel (Onco Panscan™, Genetron Health) was used to profile somatic alterations including point mutations, indels, copy number alterations, and gene fusions, as well as possible pathogenic germline variants respectively from their tumor tissues and matched genomic DNA samples.

After removing adapters and low‐quality regions with FastQC (v 0.11.2; http://www.bioinformatics.babraham.ac.uk/projects/fastqc/) and Trimmomatic (v 0.33),^12^ sequencing reads were mapped to the hg19 genome (GRch37) with BWA‐MEM (http://github.com/lh3/bwa). Somatic single‐nucleotide variants (SNVs), insertions/deletions (indels) were retrieved with MuTect (v 3.1‐0‐g72492bb; http://github.com/broadinstitute/mutect) and Strelka (v 1.0.14; http://github.com/Illumina/strelka), respectively. Germline SNVs and indels were called using Genome Analysis Toolkit (GATK, v 3.1‐0‐g72492bb).^13^ All mutations in coding regions were manually checked using Integrative Genomics Viewer (IGV, version 2.3.34).^14^ The filtered variants were annotated using Oncotator (version 1.5.1.0; http://github.com/broadinstitute/oncotator) and Variant Effect Predictor (VEP, v 83; http://github.com/Ensembl/ensembl‐vep). Copy number variations (CNVs) and structural variations (SVs) were respectively identified with in‐house modified ADTEx (http://adtex.sourceforge.net/) and CREST.^15^

### 2.3 Variant classification

We assessed the pathogenicity of germline variants based on the American College of Medical Genetics and Genomics and the Association for Molecular Pathology (ACMG/AMP) guidelines,^16^ and/or by interpretation from the ClinVar database^17^ and literature review. Somatic variants annotated as “oncogenic” or “likely oncogenic” in the OncoKB database (https://www.oncokb.org/)^18^ were classified as variants of clinical significance and used for downstream analysis.

### 2.4 Tumor mutational burden (TMB) and microsatellite instability (MSI) analysis

Similar to the MSK‐IMPACT panel, TMB was defined as somatic nonsynonymous mutation counts in coding regions per megabase (mut/Mb) of the genomic region examined. The MSI status was evaluated by the repeated percentage of 309 microsatellite loci examined, and an MSI score was established for each sample by in‐house developed scripts. Samples with an MSI score ≥ 55 were classified as MSI‐high, and those with an MSI score ≤ 35 were classified as microsatellite stable (MSS). For tumors with MSI scores between 35 and 55, those with loss of function (LOF) variants in mismatch repair (MMR) genes or TMB ≥20 mut/Mb were defined as MSI‐H, otherwise MSS.^19^

### 2.5 Statistical analysis

The chi‐squared test and Fisher's exact test were used to evaluate the association between two categorical variables. Wilcoxon's rank sum test was used to compare the distribution of continuous variables (age) between groups. Logistic regression analysis was used to establish *p* value in the case–control study. All tests were two‐sided and *p* values <0.05 were considered statistically significant. All analyses were conducted using SPSS (v 24) and R (v 4.1.3).

## 3 RESULTS

### 3.1 Major driver mutations and tumor mutational burden of Chinese PDAC patients

**FIGURE 1:**
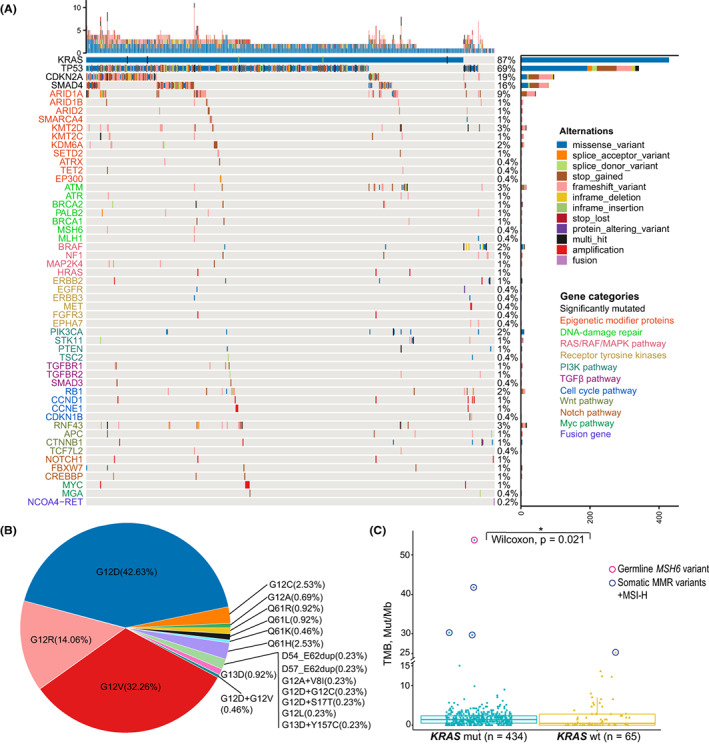
Landscape of somatic driver mutations and tumor mutational burden (TMB). (A) Summary of somatic genomic alterations and their corresponding signaling pathways in 499 Chinese PDAC patients. The majority signaling pathways altered in PDAC include RAF/RAS/MAPK, receptor tyrosine kinases, epigenetic modifiers, DNA damage repair, cell cycle, TGFβ, PI3K, Wnt, Notch, and Myc. (B) The frequency of each *KRAS* variant. (C) Tumor mutational burden landscape in *KRAS* WT (*n* = 65) and *KRAS*‐mutated (*n* = 434) PDAC cases. Patients with TMB larger than 25 mutations/Mb (*n* = 5) harbored pathogenic germline/somatic alterations in DNA mismatch repair (MMR) genes.

Overall, 499 Chinese PDAC patients diagnosed between January 2019 and December 2021 comprised the final cohort for analysis; 297 (59.5%) patients were male, and the median age at diagnosis was 60 (range 30–85) years (Table S1). The prevalence of *KRAS* mutations was 87% (434/499), similar to the TCGA study (93%, *n* = 150) and an Australian PDAC cohort (92%, *n* = 456)^9^, ^10^ (Figure 1A). The top three *KRAS* mutations were G12D (42.6%), G12V (32.3%), and G12R (13.8%), and the targetable *KRAS* mutation G12C had a low prevalence (2.5%; Figure 1B). We also observed two novel in‐frame *KRAS* duplications (D54_E62dup and D57_E62dup) in the switch II region. A recent study showed that in‐frame insertions in this region represent a new class of activating *RAS* variants with clinical relevance.^20^ Recurrent pathogenic/likely pathogenic (P/LP) mutations were identified in genes encoding epigenetic modifiers (>20%), including chromatin remodeling complex subunits (*ARID1A*, *ARID1B*, *ARID2*, *SMARCA4*) and histone methyltransferases/demethylases (*KMT2D*, *KMT2C*, *KDM6A*, and *KMT2B*; Figure 1A). About 9% of patients harbored P/LP mutations in *ARID1A*, which encodes a subunit of the SWI/SNF chromatin remodeling complex. Genetic depletion of *ARID1A* in the pancreas of mice led to pancreatic atrophy and cysts.^21^ When combined with *KRAS* activation, loss of *ARID1A* in the pancreas accelerated the formation of intraductal pancreatic mucinous neoplasms (IPMNs) and pancreatic cancer.^21^, ^22^ Overall 5.4% of patients had P/LP mutations in Wnt signaling pathway genes, including *RNF43* (3%), *APC* (1%), and *CTNNB1* (1%). Similar to *ARID1A*, the inactivation of *RNF43* in combination with *KRAS* activation in the pancreas of mice significantly increased the incidence of high‐grade cystic lesions and pancreatic cancer.^23^ These genetically engineered mouse model (GEMM) data, plus the high prevalence of *ARID1A*/*RNF43* mutations in our cohort and other PDAC cohorts,^10^, ^24^ established that these two genes were bona fide tumor suppressor genes for pancreatic cancer. In this cohort, all five individuals with TMB higher than 25 mutations/Mb had pathogenic mutations in MMR genes, and *KRAS*‐mutated patients had a significantly higher TMB level (*p* < 0.05) than *KRAS* wild‐type (WT) patients (Figure 1C).

### 3.2 Targetable mutations in *KRAS* wild‐type PDACs

**FIGURE 2:**
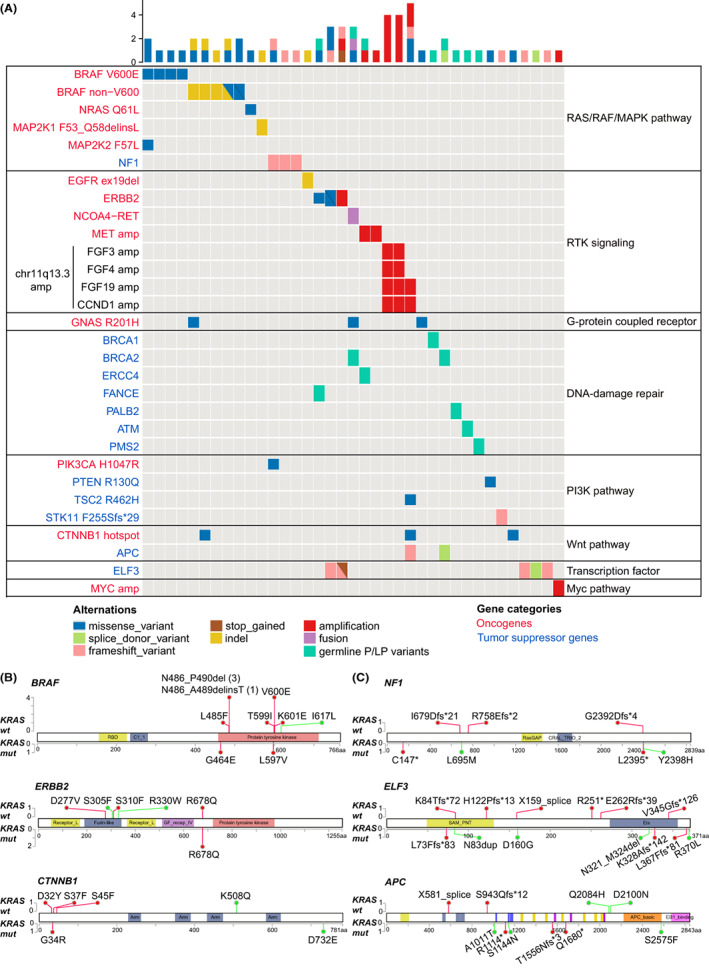
Alternative driver mutations in *KRAS* wild‐type PDAC cases. (A) Oncoplot of driver mutations and the altered signaling pathways in *KRAS* wild‐type PDAC cases. (B) *KRAS* wild‐type and *KRAS*‐mutated PDAC cases had distinct patterns of activating mutations in oncogenes *BRAF*, *ERBB2*, and *CTNNB1*. Activating mutations and variants of unknown significance (VUSs) were marked in red and green, respectively. (C) *KRAS* wild‐type and *KRAS*‐mutated PDAC cases had different patterns of inactivating mutations in tumor suppressor genes *APC*, *ELF3*, and *NF1*. Inactivating mutations and variants of unknown significance (VUSs) were marked in red and green, respectively.

In this Chinese PDAC cohort, *KRAS* mutations were mutually exclusive with P/LP alterations in *BRAF*, *CTNNB1*, *ELF3*, *FGF19*, and other cancer‐related genes (Figure S1). We observed an enrichment of alternative driver mutations in *KRAS* WT PDAC patients, some of which are actionable. For instance, activating *BRAF* mutations were significantly enriched in the *KRAS* WT group than in the *KRAS‐*mutated group (13.8% vs. 0.5%, *p* < 0.001). The activating *BRAF* mutations in *KRAS* WT patients include missense mutations V600E (*n* = 4), L485F, T599I, K601E, in‐frame deletions N486_P490del (*n* = 3) and N486_A489delinsT (Figure 2A,B). The class I *BRAF* V600E mutant functions as a monomer and is sensitive to BRAF inhibitors (BRAFi) vemurafenib, dabrafenib, and encorafenib.^25^ However, class II and class III BRAF mutants have different activation mechanisms than V600E and are generally resistant to BRAFi. For instance, the kinase activity of class II *BRAF* mutant K601E and in‐frame BRAF deletions mutants were dimer‐dependent and resistant to vemurafenib.^25^ Interestingly, *BRAF* deletion mutants were partially sensitive to BRAFi dabrafenib, and one PDAC patient with *BRAF* N486_P490del mutation achieved a partial response with dabrafenib therapy.^4^ Two individuals with compound *BRAF* mutations (K601E/T599I and N486_A489delinsT/L485F) were good candidates for the clinical trial of pan‐BRAF inhibitor KIN‐2787 (NCT04913285). We also observed oncogenic mutations in other RAF/RAS/MAPK pathway genes. Three individuals carried activating hotspot mutations in *NRAS* (Q61L), *MAP2K1* (F53_Q58delinsL), and *MAP2K2* (F57L; Figure 2A). *MAP2K1* and *MAP2K2* encode MAPK kinases MEK1 and MEK2, respectively. Three patients had inactivating mutations in *NF1*, a negative regulator of the RAS/PAF/MAPK pathway. These patients were eligible for clinical trials of MEK inhibitors (MEKi) trametinib and selumetinib (Figure 2A,C).

In *KRAS* WT patients, we also observed oncogenic alterations in receptor tyrosine kinases (RTKs) genes *EGFR*, *ERBB2*, *MET*, *RET*, and amplification of the chromosomal fragment chr11q13.3 covering fibroblast growth factors gene *FGF3*/*4*/*19* and the cell cycle gene *CCND1* (Figure 2A,B). Interestingly, one individual carried an *EGFR* exon 19 deletion mutation (L747_A750delinsP) susceptible to EGFR inhibitor erlotinib.^26^ Three patients harbored activating *ERBB2*/*HER2* mutations (S310F and R678Q) or high‐level *ERBB2/HER2* amplification (fold changes >10), which were eligible for HER2‐targeted therapy.^27^ Additionally, patients with *MET* amplification and the *NCOA4‐RET* fusion could be targeted with MET inhibitor capmatinib and RET inhibitor selpercatinib/pralsetinib, respectively. Of note, driver events in the RTK and RAF/RAS/MAPK pathway were mutually exclusive, except for one individual with concurring *BRAF* V600E and *MAP2K2* F57L mutations.

Aside from the RAF/RAS/MAPK and RTK signaling pathways, we also observed pathogenic genomic alterations in genes involved with DNA damage repair (DDR), PI3K/mTOR, and Wnt pathways (Figure 2A–C). Three patients had pathogenic germline mutations in core homologous recombination repair genes *BRCA1*/*2* and *PALB2*, which are sensitive to platinum and PARPi.^1^, ^7^ One individual had the oncogenic *PIK3CA* H1047R mutation sensitive to alpelisib, a PIK3CA inhibitor approved to treat *PIK3CA*‐mutated advanced breast cancer.^28^ Three patients with *STK11*/*TSC2* inactivating mutations or a dominant‐negative mutation of *PTEN* (R130Q) were eligible for clinical trials of mTOR inhibitor everolimus and AKT inhibitor capivasertib, respectively.^29^, ^30^

Among Wnt signaling genes, three *KRAS* WT patients had oncogenic hot mutations in *CTNNB1* (*n* = 3), and two had inactivating mutations in *APC* (Figure 2A–C). Interestingly, five patients had inactivating *ELF3* mutations, a driver of ampullary carcinoma.^31^ Moreover, pathogenic *ELF3* mutations were significantly enriched in *KRAS* WT PDACs than *KRAS*‐mutated PDACs (7.7% vs. 0.69%, *p* = 0.001). In summary, 43.1% (28/65) of *KRAS* WT patients harbored actionable genomic alterations with targeted therapy options.

### 3.3 Pathogenic germline variants and their clinical implications

**FIGURE 3:**
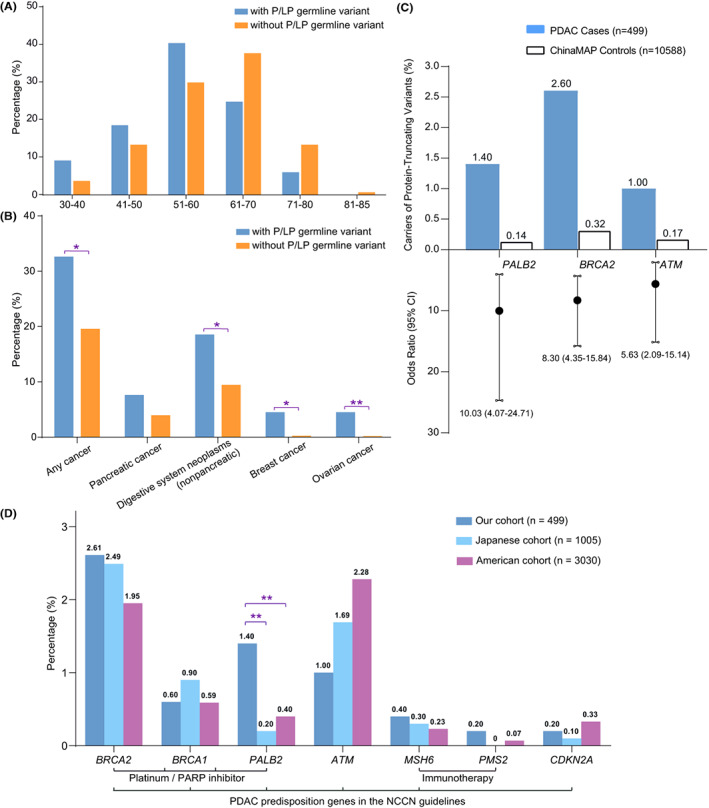
Clinical implications of pathogenic germline variants. (A) Age group distribution of PDAC patients with or without pathogenic/likely pathogenic (P/LP) germline variants. (B) The percentage of patients with or without P/LP germline variants had a family history of “Pancreatic cancer”, “Digestive system neoplasms (nonpancreatic)”, “Breast cancer”, “Ovarian cancer”, and “Any cancer (all cancer type included).” (C) Percentages of PDAC cases and controls who were carriers of protein‐truncating variants in *PALB2*, *BRCA2*, *ATM*. The genes are listed in order of decreasing odds ratios for PDAC overall. (D) The frequency of PDAC predisposition gene variants in three PDAC cohorts (Chinese, Japanese, American). Values shown above the bars refer to the percentage of patients with P/LP germline variants in a specific gene.

Pathogenic germline variants had important clinical implications for PDAC patients, including personalized therapy options and cancer surveillance.^2^, ^4^ In this Chinese PDAC cohort, we found 65 PGVs in 64 (12.8%) patients (Table S2). Among them, nine (*ATM* c.C259 > T, *ATM* c.4219del, *BRCA1* c.2149dup, *BRCA2* c.5271_5272dup, *MSH6* c.409_418del, *PALB2* c.1407_1408delinsG, *PALB2* c.2713C > T, *PALB2* c.2406 T > A, *RAD51D* c.C184C > T) were novel targetable PGVs not recorded in ClinVar (Table 1).^17^ One patient carried a familial melanoma predisposing germline variant *CDKN2A* R99P, annotated as likely pathogenic in ClinVar (Figure S2).^32^ However, it should be classified as pathogenic (≥2 Strong) according to the ACMG‐AMP guideline.^16^ Patients with a PGV tended to be younger than those without (Figure 3A and Table S1). The median age of PGV‐positive patients was lower than PGV‐negative patients (56 vs. 61 years, *p* = 0.001). Compared with PGV‐negative patients, PGV‐positive patients had significantly higher frequencies of family history of cancers, including breast/ovarian cancer and non‐pancreatic digestive system neoplasms (Figure 3B).

**TABLE 1:** Novel truncating pathogenic germline variants in PDAC predisposition gene *ATM*, *BRCA1*, *BRCA2*, *MSH6*, *PALB2*, and *RAD51D.*

Patient ID	Age at Dx (years)	Gender	Gene	Nucleotide change	Protein change	Mutation effect
5	56	Male	*ATM*	c.4219del	p.Ile1407PhefsTer44	Frameshift deletion
6	34	Male	*ATM*	c.259C > T	p.Gln87Ter	Stopgain
10	69	Male	*BRCA1*	c.2149dup	p.Glu717GlyfsTer3	Frameshift insertion
23	46	Male	*BRCA2*	c.5271_5272dup	p.Asn1758IlefsTer20	Frameshift insertion
40	58	Female	*MSH6*	c.409_418del	p.Ser137GlyfsTer9	Frameshift deletion
45	57	Male	*PALB2*	c.1407_1408delinsG	p.Cys469TrpfsTer16	Frameshift insertion
47	47	Male	*PALB2*	c.2713C > T	p.Gln905Ter	Stopgain
48	62	Male	*PALB2*	c.2406 T > A	p.Cys802Ter	Stopgain
53	80	Female	*RAD51D*	c.184C > T	p.Gln62Ter	Stopgain

For PDAC risk gene association analysis, we obtained reference control data from the China Metabolic Analytics Project (ChinaMAP) and the Genome Aggregation Database (gnomAD) non‐cancer East Asian population.^33^, ^34^ Protein‐truncating variants in three DNA damage repair genes (*PALB2*, *BRCA2*, and *ATM*) were associated with a significant risk of pancreatic cancer (*p* < 0.05), with odds ratio ranging from 5.03 to 10.03 (Figure 3C and Table S3).

To compare our results with previous studies in other ethnical groups, we extracted PDAC‐predisposing germline mutation data from one Japanese PDAC cohort (*n* = 1005) and one American PDAC cohort from the Mayo Clinic (*n* = 3030).^35^, ^36^ Of note, 95.6% (2896/3030) of patients in the American cohort were non‐Hispanic White. In our cohort, pathogenic variants in seven established pancreatic cancer‐predisposition genes (*ATM*, *BRCA1*, *BRCA2*, *CDKN2A*, *MSH6*, *PALB2*, and *PMS2*) were found in 6.4% (95% confidence interval [CI], 4.25–8.55) of case‐patients versus 1.7% (95% CI, 1.60–1.87) of controls (Table S3).^37^ The most common PGVs were *BRCA2* (2.6%; 95% CI, 1.20–4.00), *PALB2* (1.4%; 95% CI, 0.37–2.44), and *ATM* (1.0%; 95% CI, 0.13–1.88; Figure 3D). *PALB2* PGVs were statistically more frequent in Chinese patients than in American patients (*p* < 0.01), while *ATM* PGVs showed the opposite trend (Figure 3D). Interestingly, a recent study also showed that *PALB2* PGVs were more prevalent in a Chinese PDAC cohort than in an American PDAC cohort from the Johns Hopkins Hospital (6/1009 [0.6%] vs. 2/854 [0.2%]), although the difference was not statistically significant.^11^

### 3.4 Pathogenic germline and somatic mutations in DNA damage repair genes

**FIGURE 4:**
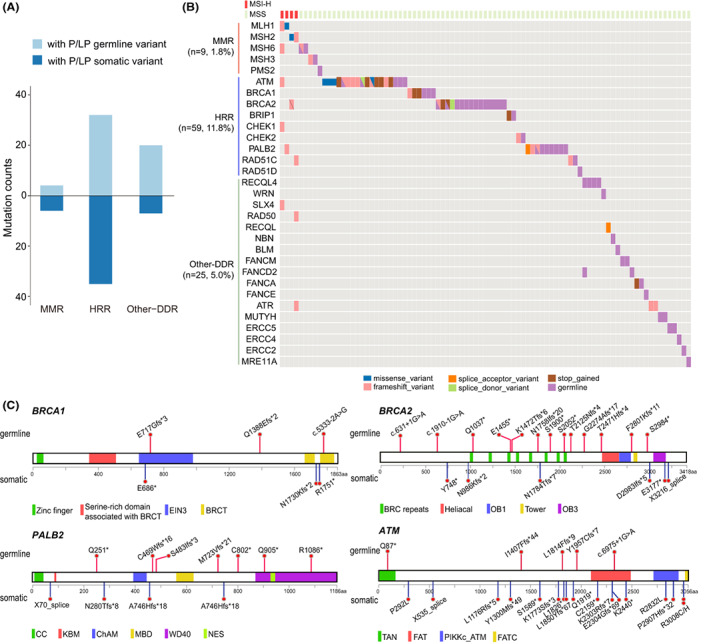
Pathogenic/likely pathogenic (P/LP) variants in DNA damage repair genes. (A) Number of P/LP germline (upper) and somatic (below) variants in mismatch repair (MMR), homologous recombination repair (HRR), and other DNA damage repair (DDR) genes. (B) Oncoplot of DDR gene mutations, microsatellite instability (MSI) status, and the altered signaling pathways in PDAC cases. (C) Pathogenic/likely pathogenic germline and somatic variants in targetable HRR genes *ATM*, *BRCA1*, *BRCA2*, and *PALB2*.

The NCCN guidelines recommended olaparib and pembrolizumab for advanced PDAC patients with germline *BRCA1/2* (g*BRCA*) mutations and mismatch repair deficiency (dMMR)/microsatellite instability (MSI), respectively.^2^ These recommendations made defects in DNA damage repair (DDR) an attractive target for PDAC precision therapy. Consistently, the prevalence of DDR gene PGVs (86%, 56/65) in our cohort is much higher than non‐DDR gene PGVs (14%, 9/65; Figure S2). Therefore, we profiled the pathogenic germline and somatic variants of DDR genes in this Chinese PDAC cohort. There was a significant enrichment of DDR gene pathogenic/likely pathogenic (P/LP) mutations in the homologous recombination repair (HRR) pathway than in other DDR pathways (Figure 4A,B). About 0.8% (4/499) of this cohort was MSI‐H (Figure 4B), similar to the prevalence of dMMR in the Memorial Sloan Kettering (MSK) Cancer Center PDAC cohort (0.8%, 7/833).^3^ Interestingly, all MSI‐H patients in the cohort carried somatic MMR gene variants, but all dMMR patients in the MSK cohort had germline MMR variants.^3^

To investigate the therapeutic potential of HRR gene mutations, we selected 14 genes (*ATM*, *BARD1*, *BRCA1*, *BRCA2*, *BRIP1*, *CDK12*, *CHEK1*, *CHEK2*, *FANCL*, *PALB2*, *RAD51B*, *RAD51C*, *RAD51D*, and *RAD54L*), which were biomarkers for the prescription of olaparib for patients with metastatic castration‐resistant prostate cancer.^38^ This gene list shared eight genes with the 17 HRR genes used by O'Reilly and colleagues to define *BRCA1/2* and *PALB2* as core HRR genes in PDAC.^39^ Mutations in core HRR genes resulted in higher genomic instability in PDAC than mutations in non‐core HRR genes.^39^ Consistently, results of a phase 2 trial demonstrated that PARPi rucaparib had antitumor activity in PDAC patients with germline or somatic mutations in core HRR genes, including somatic mutations in *BRCA2* and germline mutations in *PALB2*.^40^ In this PDAC cohort, the prevalence of *BRCA* PGVs was 3.2% (95% CI, 1.66–4.76), and the combined prevalence of germline/somatic mutations in *BRCA* and *PALB2* was 8.4% (95% CI, 5.97–10.86), indicating the potential of expanded PARPi therapy in PDAC (Figure 4B,C). A clinical trial of ATR kinase inhibitor celarasertib (AZD6738) showed that solid tumors with *ATM* mutation were sensitive to ATR inhibition.^41^ Interestingly, 1.0% (95% CI, 0.13–1.88) of patients in this cohort harbored pathogenic mutations in *ATM* (Figure 4B,C), which may be eligible for the clinical trials of celarasertib.

### 3.5 Characteristics of Chinese early‐onset pancreatic cancer patients

**FIGURE 5:**
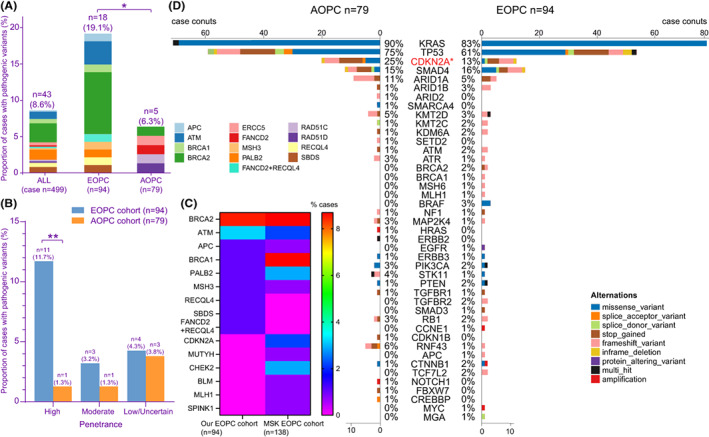
Pathogenic genomic variants (PGVs) in early‐onset pancreatic cancer (EOPC). (A) Pathogenic germline variants were enriched in early‐onset pancreatic cancer (EOPC, ≤50 years) than average‐onset pancreatic cancer (APOC, ≥70 years). (B) PGVs with high penetrance were enriched in EOPC than APOC. (C) Heatmap of PGV frequency in the Chinese EOPC cohort (*n* = 94) and the Memorial Sloan Kettering Cancer Center (MSK) EOPC cohort (*n* = 138). (D) Pathogenic/likely pathogenic somatic mutations in EOPC cohort (*n* = 94) and AOPC cohort (*n* = 79).

Accumulating evidence showed that there was a rising incidence of PDAC in younger individuals, or early‐onset pancreatic cancer (EOPC).^42^, ^43^, ^44^ To explore precision therapy opportunities for this patient subgroup with unmet clinical needs, we analyzed the EOPC subgroup of our cohort (*n* = 94, diagnosis age of 50 or younger). Fifty (53.2%) patients were male, and the median age at diagnosis was 46 (range 30–50). Nineteen PGVs were identified in 18 (19.1%) EOPC patients. PGVs including high‐penetrance PGVs were significantly enriched in EOPC than in average‐onset pancreatic cancer (AOPC; ≥70 years; 19.1% vs. 6.3%, *p* = 0.014; 11.7% vs. 1.27%, *p* = 0.007; Figure 5A,B). The most frequent PGV in this Chinese EOPC cohort was *BRCA2*, similar to data of the MSK EOPC cohort (Figure 5C).^45^ In the EOPC subgroup, there were three patients with *BRAF* V600E mutation and one with *EGFR* exon 19 deletion (L747_A750delinsP) mutation, which were absent in the AOPC subgroup (Figure 5D). The prevalence of somatic *CDKN2A* mutations in EOPC was significantly lower than in AOPC (*p* < 0.05), consistent with data from a combined analysis of four PDAC cohorts in North America (COMPASS, ICGC, POG, TCGA; Figure 5D).^46^

## 4 DISCUSSION

With the approval of PARP/NTRK/PD‐1 inhibitors, the treatment of PDAC finally entered the era of precision therapy. However, the low prevalence of g*BRCA* variants, *NTRK* fusion, and dMMR/MSI in PDAC patients limited the practice of precision medicine.^4^ Therefore, there is an urgent need to identify new therapeutic targets.

Multiple studies have established *KRAS* mutations as the dominant driver for PDAC.^8^, ^9^, ^10^ However, direct targeting of KRAS has proved very difficult.^4^ Recently, two KRAS G12C inhibitors, sotorasib (AMG 510) and adagrasib (MRTX 849), showed excellent anti‐tumor activity in lung cancer. In the CodeBreaK100 trial, sotorasib achieved an objective response rate (ORR) of 36% in patients with *KRAS* G12C‐mutated metastatic non‐small cell lung cancer (NSCLC).^5^ Similarly, in the KRYSTAL‐1 study, adagrasib achieved an ORR of 42.9% in pretreated *KRAS* G12C‐mutated NSCLC patients.^6^ New data from the phase 1/2 CodeBreaK100 and KRYSTAL‐1 trials demonstrated that both sotorasib and adagrasib had promising anti‐tumor activity in *KRAS* G12C‐mutated PDAC. Among 38 stage IV patients treated with sotorasib, eight (21%) had a partial response, and the disease control rate (DCR) was 84%.^47^ In ten *KRAS* G12C‐mutated patients treated with adagrasib, five (50%) achieved a partial response, and the DCR was 100%.^48^ Interestingly, a recent report showed that T‐cell receptor (TCR) gene therapy targeting the *KRAS* G12D mutant resulted in a partial response of 72% in a treatment‐refractory metastatic pancreatic cancer patient.^49^ These results indicated that new KRAS‐targeting agents like sotorasib, adagrasib, and TCR‐T gene therapy could shift the treatment paradigm of *KRAS*‐mutated PDAC in the future.

Aside from *KRAS*, oncogenic mutations in other RAS/RAF/MAPK pathway genes also can drive pancreatic cancer.^4^ For instance, three out of the ten *KRAS* WT patients in the TCGA PDAC cohort harbored activating *BRAF* alterations, including two in‐frame deletions (N486_P490del and N486_A489 > K) and one *CUX1*‐*BRAF* fusion.^10^ In this Chinese cohort, activating *BRAF* mutations were significantly enriched in the *KRAS* WT group compared with the *KRAS*‐mutated group (13.8% vs. 0.5%, *p* < 0.001). Except for four individuals with the class I V600E mutation, all *BRAF*‐mutated patients harbored non‐V600 mutations, including missense mutations (L485F, T599I, K601E) and in‐frame deletions (N486_P490del and N486_A489delinsT). Two *KRAS* WT patients had hotspot mutations in *MAP2K1* and *MAP2K2*, which encode the MAPK kinase MEK1 and MEK2, respectively. Our results indicated that *KRAS* WT PDAC patients driven by oncogenic mutations in the RAS/RAF/MAPK signaling pathway could participate in the basket clinical trials of BRAFi and MEKi.

Genetic defects in the DNA damage response (DDR) pathway often result in genome instability.^4^ DDR defects is an attractive target for cancer precision therapy. However, only a small subset of PDAC patients can benefit from immunotherapy due to the low prevalence of dMMR/MSI in PDAC.^4^ A retrospective analysis of 8323 patients with PDAC showed that only 1%–2% was dMMR/MSI.^50^ Consistently, only 0.8% (4/499) of this Chinese PDAC cohort was MSI‐H. Compared to MSI/dMMR, homologous recombination repair deficiency (HRD) is a more attractive therapeutic target due to its higher prevalence in PDAC.^51^ Recently, O'Reilly and colleagues reported that 19% (50/262) of a PDAC cohort at MSK had HRD (15% germline and 4% somatic).^39^ Among 17 HRR genes, more patients had variants in core‐HRR genes *BRCA1, BRCA2*, and *PALB2* (12%) than in 14 non‐core HRR genes (7%). Similarly, in this Chinese cohort, 11.8% (59/499) of patients had mutations in HRR genes, including 6.4% in core‐HRR genes and 5.4% in non‐core HRR genes. Although the POLO trial only included PDAC patients with germline *BRCA1*/*2* mutations, a retrospective study showed that germline and somatic *BRCA1*/*2* had similar actionability to PARP inhibitors.^52^ Consistently, results of a phase 2 trial showed that PARPi rucaparib was effective in PDAC patients with germline or somatic *BRCA*/*PALB2* variants.^40^ These data indicated that PARPi therapy in pancreatic cancer could be expanded from germline *BRCA* mutations to germline or somatic mutations of *BRCA*/*PALB2*.^4^ We also observed that 3.8% of patients had pathogenic *ATM* mutations. *ATM* was a novel therapeutic target due to the proposed synthetic lethality with ATR inhibition.^53^ Consistently, data of a phase 1 trial (NCT02264678) showed that ATR kinase inhibitor ceralasertib plus carboplatin achieved confirmed partial response in two patients with advanced solid tumors, which had no or low ATM nuclear staining.^41^ All these results provided compelling evidence that DDR defects are promising targets for PDAC precision therapy.^4^

The rising incidence of early‐onset pancreatic cancer (EOPC) is concerning.^42^, ^44^, ^45^, ^46^ Due to the limited knowledge of targetable mutations in this uncommon disease, the practice of precision medicine in EOPC was difficult. One study at MSK reported that EOPC (*n* = 95; ≤55 years) patients had higher frequency of *SMAD4* mutations than patients with average‐onset pancreatic cancer (AOPC; *n* = 203, ≥70 years).^42^ In a combined analysis of four PDAC cohorts in North America (COMPASS, ICGC, POG, TCGA), Tsang et al. observed a lower frequency of *CDKN2A* mutations in EOPC (*n* = 117; ≤55 years) than AOPC (*n* = 165, ≥70 years) but no difference of *SMAD4* mutation frequency in these two groups.^46^ O'Reilly and colleagues at MSK recently conducted genomic profiling of a large EOPC cohort (*n* = 450, ≤50 years; 132 and 138 underwent somatic and germline testing, respectively) and identified multiple actionable genomic alterations including dMMR, *IDH1* R132C mutation, *NTRK*/*NRG1* fusions, and germline *BRCA* mutations.^45^ Of note, the above EOPC cohorts were in North America, and the majority of patients were White.^42^, ^44^, ^45^, ^46^ In this Chinese PDAC cohort, the frequency of P/LP *CDKN2A* mutation was significantly lower in EOPC than in AOPC, and there was no difference in P/LP *SMAD4* mutation frequency in these two subgroups, similar to the results of Tsang.^46^ *BRCA2* was the most common germline alteration in the Chinese EOPC cohort, similar to the large MSK EOPC cohort.^45^ For targetable somatic mutations, we observed MSI, *EGFR* exon 19 deletion, and *BRAF* V600E mutation in this Chinese EOPC cohort, distinct from data of the large MSK EOPC cohort.^45^ Our work, together with previous EOPC genomic profiling studies, suggested that the identification of actionable mutations in EOPC patients through germline/somatic testing could provide the opportunity for precision therapy.

Accumulating evidences indicated that specific genomic alterations and *KRAS* variant allele frequencies could affect the survival of PDAC patients. In a large Japanese PDAC cohort study (*n* = 1162), *KRAS* variant allele frequency (VAF) was inversely associated with disease‐free survival (DFS) and overall survival (OS).^54^ A retrospective study of 111 PDAC patients showed that patients with *KRAS* G12R versus non‐G12R mutations had significantly longer OS (HR 0.55) and PFS (HR 0.58), respectively.^55^ Another retrospective study of 587 PDAC patients revealed that patient with more than one driver mutations had worse OS than those with one driver mutation (18.2 vs. 32.3 months, *p* = 0.033).^56^ These results suggested that genomic profiling could provide important predictive information on the survival of PDAC patients.

In addition to systemic therapy decision‐making, PGVs also have substantial implications for cancer surveillance and risk management. A 20‐year pancreatic cancer surveillance study of 347 *CDKN2A* PGV carriers identified 36 PDAC cases in 31 (8.9%) individuals through annual magnetic resonance imaging.^57^ Among these PDAC cases, 83.3% (30/36) were resectable at the time of imaging and 33.3% (12/36) were stage 1. This work indicated that surveillance in the high‐risk population with PDAC predisposing PGVs could improve patient outcomes with early detection and timely resection.

Overall, our cohort and the TCGA cohort had a similar prevalence of *KRAS* mutations (87% vs. 93%).^10^ Compared to the TCGA cohort, this Chinese PDAC cohort had a higher prevalence of G12V (32.3% vs. 22.4%) but a lower prevalence of G12R (14.1% vs. 20%), which is consistent with another Chinese PDAC cohort study.^58^ We also compared germline *BRCA1/2* variants of our cohort with those of the phase 3 POLO trial cohort (*n* = 2206).^59^ No Ashkenazi Jewish–specific *BRCA2* variants (6174delT) or *BRCA1* variants (187delAG, 5382insC, and 5385insC) were found in this Chinese cohort. These results suggested that pathogenic somatic and germline variants in PDAC patients are associated with race and ethnicity.

## 5 CONCLUSION

In conclusion, at least 20.4% of Chinese PDAC patients in this study harbored actionable genomic alterations. *PALB2*, *BRCA2*, and *ATM* PGVs were associated with high risks for pancreatic cancer in the Chinese population. For the uncommon subgroup of *KRAS* WT and EOPC patients, genomic profiling results could benefit them with the opportunity of precision therapy and genomically‐matched clinical trials.

## AUTHOR CONTRIBUTIONS

**Zhiming Zhao:** Conceptualization (equal); formal analysis (equal); investigation (equal); resources (equal); validation (equal). **Xiaomo Li:** Conceptualization (equal); formal analysis (lead); investigation (equal); methodology (equal); visualization (lead); writing – original draft (lead). **Fei Wang:** Data curation (equal); formal analysis (supporting); investigation (supporting); methodology (equal); resources (supporting); validation (supporting). **Yong Xu:** Data curation (equal); formal analysis (supporting); investigation (equal); resources (equal); validation (equal). **Si Liu:** Data curation (supporting); formal analysis (supporting); investigation (supporting); methodology (supporting); validation (supporting). **Quanli Han:** Data curation (supporting); investigation (supporting); resources (supporting); validation (supporting). **Zhiying Yang:** Formal analysis (supporting); resources (supporting); validation (supporting). **Weiwei Huang:** Investigation (supporting); methodology (supporting); validation (supporting). **Zhuzeng Yin:** Data curation (supporting); investigation (supporting); resources (supporting); validation (supporting). **Qu Liu:** Data curation (supporting); resources (supporting); validation (supporting). **Haidong Tan:** Formal analysis (supporting); validation (supporting). **Tonghui Ma:** Supervision (supporting); validation (supporting); writing – review and editing (supporting). **Shuang Si:** Investigation (supporting); resources (supporting); validation (supporting). **Jia Huang:** Formal analysis (supporting); methodology (supporting); validation (supporting). **Hongling Yuan:** Investigation (supporting); methodology (supporting); visualization (supporting). **Wei Li:** Data curation (supporting); validation (supporting). **Rong Liu:** Conceptualization (lead); investigation (equal); project administration (lead); resources (lead); supervision (lead).

## FUNDING INFORMATION

This research did not receive any specific grant from funding agencies in the public, commercial, or not‐for‐profit sectors.

## CONFLICT OF INTEREST STATEMENT

Xiaomo Li, Si Liu, Weiwei Huang, Tonghui Ma, Hongling Yuan, and Wei Li are employees of Hangzhou Jichenjunchuang Medical Laboratory, Co. Ltd., Hangzhou, China. The remaining authors declare that the research was conducted in the absence of any commercial or financial relationships that could be construed as a potential conflict of interest.

## ETHICS STATEMENT

The study was approved by the Ethical Committee of the Chinese People's Liberation Army (PLA) General Hospital and the study participants gave written informed consent.

## Supporting information


Figure S1–S2

Table S1–S3

## Data Availability

The data that support the findings of this study are available from the corresponding author upon reasonable request.
