# Supplementary material for: Pathogenic genomic alterations in Chinese pancreatic cancer patients and their therapeutical implications
Source: Cancer Med. 2023 Mar 31;12(10):11672–85. doi: 10.1002/cam4.5871 (PMC10242355; doi:10.1002/cam4.5871)

Supplementary Fig. 1: Co-occurring and mutually exclusive pathogenic/likely pathogenic somatic gene alteration patterns in this Chinese PDAC cohort.


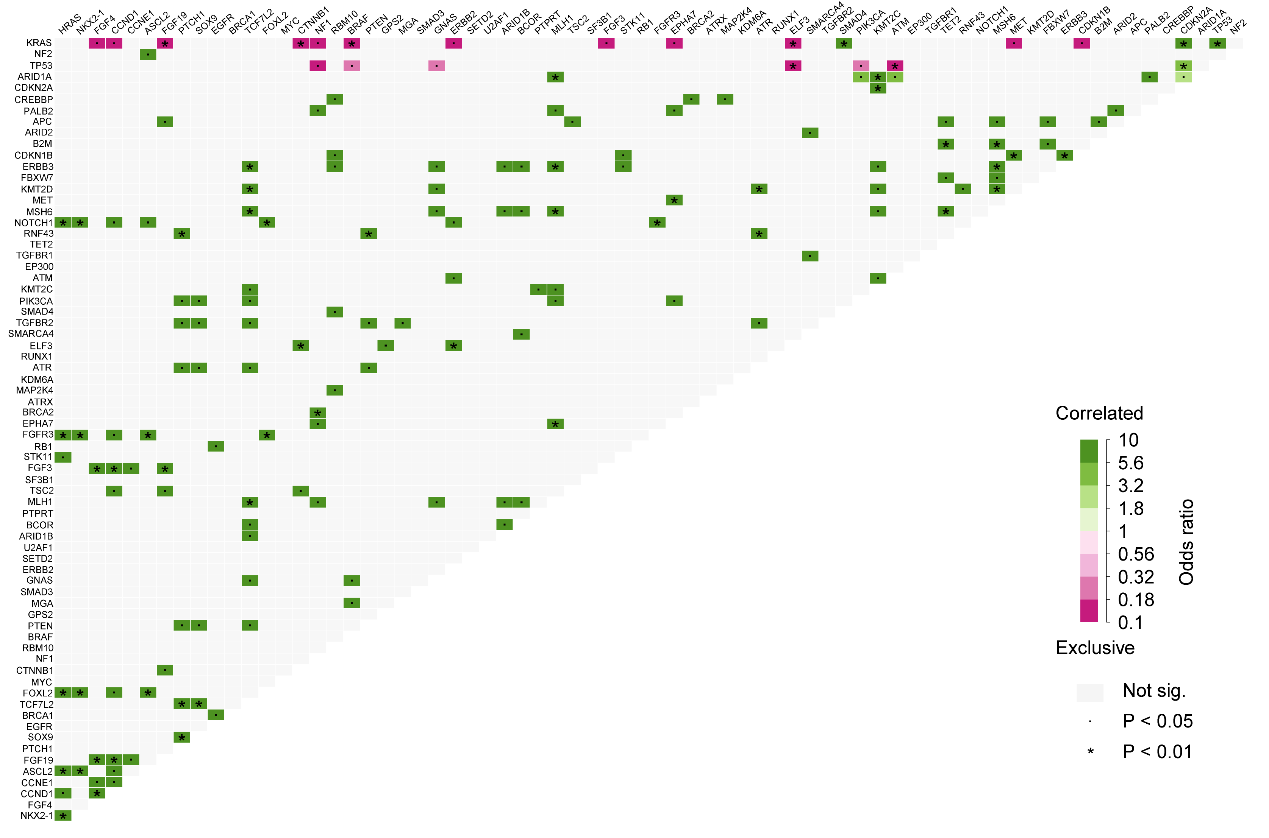


Supplementary Fig. 2: The distribution and numbers of pathogenic/likely pathogenic germline variants in MMR, HRR and other-DDR pathways and six non-DDR genes in this Chinese PDAC cohort.


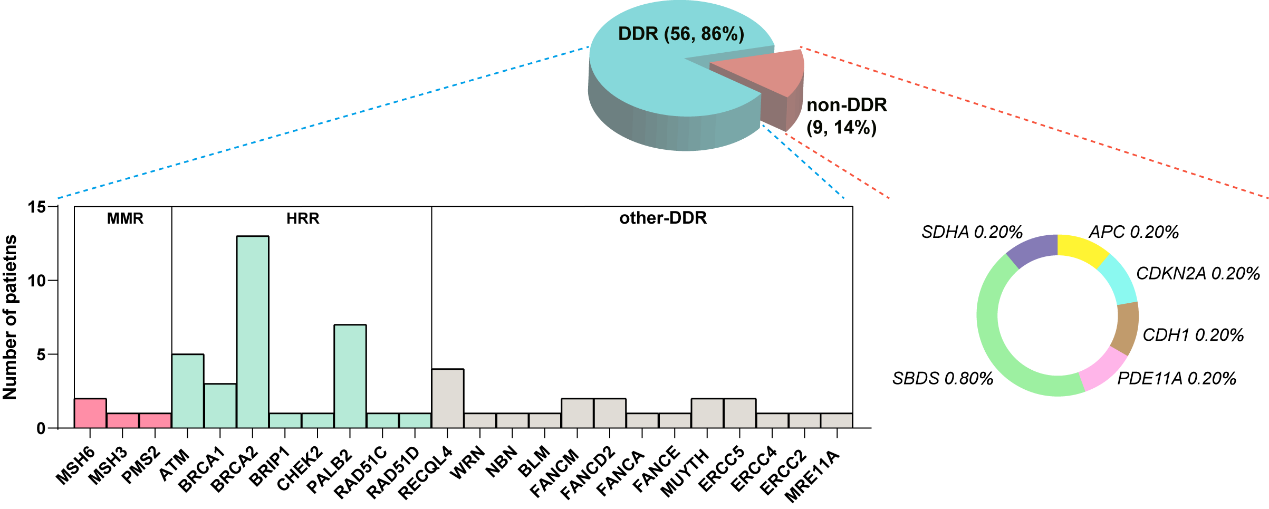

Supplement: Supplementary file 1 — Figure S1–S2 [file CAM4-12-11672-s001.docx]
