# Supplementary material for: Pathogenic genomic alterations in Chinese pancreatic cancer patients and their therapeutical implications
Source: Cancer Med. 2023 Mar 31;12(10):11672–85. doi: 10.1002/cam4.5871 (PMC10242355; doi:10.1002/cam4.5871)
Supplement: Supplementary file 2 — Table S1–S3 [file CAM4-12-11672-s002.docx]

Table S1 Demographic and clinical characteristics of this Chinese PDAC cohort

| **Clinical**  **characteristics** | **No. of**  **patents (%)**  **(n = 499)** | **No. of patents (%)** | | **P value** |
| --- | --- | --- | --- | --- |
|  |  | **Deleterious**  **mutation**  **=No (n=435)** | **Deleterious**  **mutation**  **=Yes (n=64)** |  |
| **Age at diagnosis, y** | | | | |
| **Median (range)** | 60 (30-85) | 61 (35-85) | 56 (30-80) | 0.001 |
| **≤40** | 23 (4.6%) | 17 (3.9%) | 6 (9.4%) | 0.001 |
| **41-50** | 71 (14.2%) | 59 (13.6%) | 12 (18.8%) |  |
| **51-60** | 157 (31.5%) | 131 (30.1%) | 26 (40.6%) |  |
| **61-70** | 181 (36.3%) | 165 (37.9%) | 16 (25.0%) |  |
| **71-80** | 63 (12.6%) | 59 (13.6%) | 4 (6.3%) |  |
| **>80** | 4 (0.8%) | 4 (0.9%) | 0 (0.0%) |  |
| **≤50** | 94 (18.8%) | 76 (17.4%) | 18 (28.6%) | 0.058 |
| **≤55** | 102 (20.4%) | 76 (17.4%) | 26 (40.6%) | <0.0001 |
| **Gender** | | | | |
| **Male** | 297 (59.52%) | 261 (60.00%) | 36 (56.25%) | 0.568 |
| **Female** | 202 (40.48%) | 174 (40.00%) | 28 (43.75%) |  |
| **Family history** | | | | |
| **Any cancer** | 107 (21.44%) | 86 (19.77%) | 21 (32.81%) | 0.022 |
| **Pancreatic cancer** | 23 (4.61%) | 18 (4.14%) | 5 (7.81%) | 0.199 |
| **Digestive system neoplasms (non-pancreatic)** | 54 (10.82%) | 42 (9.66%) | 12 (18.75%) | 0.049 |
| **Breast cancer** | 5 (1.00%) | 2 (0.46%) | 3 (4.69%) | 0.017 |
| **Ovarian cancer** | 4 (0.80%) | 1 (0.23%) | 3 (4.69%) | 0.007 |

Wilcoxon’s rank sum test and Point-biserial correlation analysis for age at diagnosis, *chi*-squared test and Fisher’s exact test for others.

Table S2 Germline pathogenic variants in cancer predisposition genes

| Patient ID | Age at Dx (Years) | Gender | Gene | Nucleotide change | Protein change | Mutation Effect | dbSNP ID | ClinVar interpretation |
| --- | --- | --- | --- | --- | --- | --- | --- | --- |
| 1 | 48 | Male | APC | c.509_512del | p.Asp170ValfsTer4 | Frameshift deletion | rs387906231 | Pathogenic |
| 2 | 30 | Male | ATM | c.6975+1G>A | N/A | Splicing | — | Likely pathogenic |
| 3 | 54 | Female | ATM | c.5441dup | p.Leu1814PhefsTer9 | Frameshift insertion | rs1555106508 | Pathogenic |
| 4 | 33 | Male | ATM | c.5870_5871del | p.Tyr1957CysfsTer7 | Frameshift deletion | rs1060501657 | Pathogenic |
| 5 | 56 | Male | ATM | c.4219del | p.Ile1407PhefsTer44 | Frameshift deletion | — | — |
| 6 | 34 | Male | ATM | c.259C>T | p.Gln87Ter | Stopgain | — | — |
| 7 | 62 | Male | BLM | c.320dup | p.Leu107PhefsTer36 | Frameshift insertion | rs781221411 | Pathogenic/Likely pathogenic |
| 8 | 50 | Male | BRCA1 | c.5333-2A>G | N/A | Splicing | rs397509264 | Likely pathogenic |
| 9 | 57 | Male | BRCA1 | c.4161_4162del | p.Gln1388GlufsTer2 | Frameshift deletion | rs80357565 | Pathogenic |
| 10 | 69 | Male | BRCA1 | c.2149dup | p.Glu717GlyfsTer3 | Frameshift insertion | — | — |
| 11 | 71 | Male | BRCA2 | c.1910-1G>A | N/A | Splicing | rs1566225769 | Pathogenic/Likely pathogenic |
| 12 | 48 | Male | BRCA2 | c.3109C>T | p.Gln1037Ter | Stopgain | rs80358557 | Pathogenic |
| 13 | 55 | Female | BRCA2 | c.4363G>T | p.Glu1455Ter | Stopgain | rs1566230144 | Pathogenic |
| 14 | 38 | Male | BRCA2 | c.4415_4418del | p.Lys1472ThrfsTer6 | Frameshift deletion | rs397507333 | Pathogenic |
| 15 | 45 | Female | BRCA2 | c.8951C>G | p.Ser2984Ter | Stopgain | rs80359146 | Pathogenic |
| 16 | 56 | Female | BRCA2 | c.7409dup | p.Thr2471HisfsTer4 | Frameshift insertion | rs397507915 | Pathogenic |
| 17 | 47 | Female | BRCA2 | c.5699C>A | p.Ser1900Ter | Stopgain | rs397507797 | Pathogenic |
| 18 | 40 | Female | BRCA2 | c.631+1G>A | N/A | Splicing | rs81002897 | Pathogenic/Likely pathogenic |
| 19 | 59 | Male | BRCA2 | c.8400_8402delinsAAAA | p.Phe2801LysfsTer11 | Frameshift block substitution | rs483353077 | Pathogenic |
| 20 | 61 | Male | BRCA2 | c.6373dup | p.Thr2125AsnfsTer4 | Frameshift insertion | [rs80359577](https://www.ncbi.nlm.nih.gov/snp/rs80359577) | Pathogenic |
| 21 | 50 | Male | BRCA2 | c.6816_6820del | p.Gly2274AlafsTer17 | Frameshift deletion | [rs587781803](https://www.ncbi.nlm.nih.gov/snp/rs587781803) | Pathogenic |
| 22 | 48 | Female | BRCA2 | c.6155C>G | p.Ser2052Ter | Stopgain | [rs786202461](https://www.ncbi.nlm.nih.gov/snp/rs786202461) | Pathogenic |
| 23 | 46 | Male | BRCA2 | c.5271_5272dup | p.Asn1758IlefsTer20 | Frameshift insertion | — | — |
| 24 | 57 | Female | BRIP1 | c.1343G>A | p.Trp448Ter | Stopgain | rs775171520 | Pathogenic/Likely pathogenic |
| 25 | 68 | Male | CDKN2A | c.296G>C | p.Arg99Pro | Nonsynonymous | rs754806883 | Likely pathogenic |
| 26 | 56 | Male | CDH1 | c.220C>T | p.Arg74Ter | Stopgain | rs876658932 | Pathogenic |
| 27 | 53 | Female | ERCC2 | c.594+2_594+5del | N/A | Splicing | rs762309206 | Likely pathogenic |
| 28 | 56 | Male | ERCC4 | c.55G>T | p.Glu19Ter | Stopgain | — | — |
| 29 | 56 | Female | ERCC5 | c.1173dup | p.Lys392Ter | Stopgain | rs1283214655 | Likely pathogenic |
| 30 | 76 | Male | ERCC5 | c.1173dup | p.Lys392Ter | Stopgain | rs1283214655 | Likely pathogenic |
| 31 | 70 | Female | FANCD2 | c.1546-2A>C | N/A | Splicing | — | — |
| 32 | 48 | Male | FANCD2 | c.3817C>T | p.Arg1273Ter | Stopgain | — | Pathogenic |
| 33 | 60 | Male | FANCM | c.2309_2310del | p.His770ArgfsTer8 | Frameshift deletion | — | — |
| 34 | 67 | Female | FANCM | c.2125_2126del | p.Q709VfsX7 | Frameshift deletion | — | — |
| 35 | 65 | Female | FANCE | c.355C>T | p.Gln119Ter | Stopgain | [rs121434505](https://www.ncbi.nlm.nih.gov/snp/rs121434505) | Pathogenic​ |
| 36 | 59 | Male | FANCA | c.1777-1G>C | N/A | Splicing | [rs755104393](https://www.ncbi.nlm.nih.gov/snp/rs755104393) | Likely pathogenic​ |
| 37 | 51 | Female | MRE11A | c.1540_1552dup | p.Glu518GlyfsTer4 | Frameshift insertion | — | — |
| 38 | 42 | Female | MSH3 | c.1341-2A>T | N/A | Splicing | rs750876165 | Likely pathogenic |
| 39 | 66 | Male | MSH6 | c.3775_3776del | p.Asn1259CysfsTer15 | Frameshift deletion | [rs1572745157](https://www.ncbi.nlm.nih.gov/snp/rs1572745157) | Pathogenic​ |
| 40 | 58 | Female | MSH6 | c.409_418del | p.Ser137GlyfsTer9 | Frameshift deletion | — | — |
| 41 | 60 | Female | MUTYH | c.778C>T | p.Arg260Trp | Nonsynonymous | rs773087549 | Pathogenic/Likely pathogenic |
| 42 | 54 | Female | MUTYH | c.757C>T | p.Gln253Ter | Stopgain | rs786203115 | Pathogenic |
| 43 | 63 | Male | NBN | c.565C>T | p.Gln189Ter | Stopgain | rs1198614767 | Pathogenic |
| 44 | 61 | Female | PALB2 | c.3256C>T | p.Arg1086Ter | Stopgain | [rs587776527](https://www.ncbi.nlm.nih.gov/snp/rs587776527) | Pathogenic/Likely pathogenic​ |
| 45 | 57 | Male | PALB2 | c.1407_1408delinsG | p.Cys469TrpfsTer16 | Frameshift insertion | — | — |
| 46 | 62 | Female | PALB2 | c.751C>T | p.Gln251Ter | Stopgain | rs180177091 | Pathogenic |
| 47 | 47 | Male | PALB2 | c.2713C>T | p.Gln905Ter | Stopgain | — | — |
| 48 | 52 | Male | PALB2 | c.1447_1448del | p.Ser483IlefsTer3 | Frameshift deletion | [rs1966967463](https://www.ncbi.nlm.nih.gov/snp/rs1966967463) | Pathogenic |
| 49 | 62 | Male | PALB2 | c.2406T>A | p.Cys802Ter | Stopgain | — | — |
| 50 | 61 | Female | PALB2 | c.2167_2168del | p.Met723ValfsTer21 | Frameshift deletion | [rs587776416](https://www.ncbi.nlm.nih.gov/snp/rs587776416) | Pathogenic |
| 51 | 56 | Male | PDE11A | c.985C>T | p.Arg329Ter | Stopgain | rs188985665 | Conflicting interpretations of pathogenicity​: Likely pathogenic (1); Uncertain significance (1) |
| 52 | 71 | Female | RAD50C | c.432dup | p.Pro145ThrfsTer10 | Frameshift insertion | [rs1555594590](https://www.ncbi.nlm.nih.gov/snp/rs1555594590) | Pathogenic |
| 53 | 80 | Female | RAD51D | c.184C>T | p.Gln62Ter | Stopgain | — | — |
| 54 | 38 | Female | RECQL4 | c.2768_2769del | p.Leu923AlafsTer53 | Frameshift deletion | — | — |
| 55 | 60 | Male | RECQL4 | c.957C>A | p.Tyr319Ter | Stopgain | — | — |
| 56 | 54 | Female | RECQL4 | c.2416_2420dup | p.Arg807ProfsTer38 | Frameshift insertion | — | — |
| 32 | 48 | Male | RECQL4 | c.3395-1G>A | N/A | Splicing | — | — |
| 57 | 42 | Male | SBDS | c.258+2T>C | N/A | Splicing | rs113993993 | Pathogenic/Likely pathogenic​ |
| 58 | 68 | Male | SBDS | c.258+2T>C | N/A | Splicing | rs113993993 | Pathogenic/Likely pathogenic​ |
| 59 | 52 | Male | SBDS | c.258+2T>C | N/A | Splicing | rs113993993 | Pathogenic/Likely pathogenic​ |
| 60 | 56 | Male | SBDS | c.258+2T>C | N/A | Splicing | rs113993993 | Pathogenic/Likely pathogenic​ |
| 61 | 59 | Male | SDHA | c.1534C>T | p.Arg512Ter | Stopgain | rs748089700 | Pathogenic/Likely pathogenic |
| 62 | 64 | Female | WRN | c.2959C>T | p.Arg987Ter | Stopgain | rs747319628 | Pathogenic/Likely pathogenic |
| 63 | 64 | Female | PMS2 | c.2192_2196del | p.Leu731CysfsTer3 | Frameshift deletion | [rs63750695](https://www.ncbi.nlm.nih.gov/snp/rs63750695) | Pathogenic |
| 64 | 59 | Female | CHEK2 | c.1555C>T | p.Arg519Ter | Stopgain | [rs200432447](https://www.ncbi.nlm.nih.gov/snp/rs200432447) | Pathogenic/Likely pathogenic |

Table S3 Associations between protein-truncating germline pathogenic variants in 29 genes and risk of pancreatic ductal adenocarcinoma

| **Genes** | **Cases** | **ChinaMAP Controls**  **and Cancer Risk** | | | | **gnomAD Controls (East Asia)**  **and Cancer Risk** | | | |
| --- | --- | --- | --- | --- | --- | --- | --- | --- | --- |
|  | Cases with protein-truncating variants, No. (n=499) | Controls with protein-truncating variants, No. (n=10588) | OR  (95% CI) | P Value^a^ | Adjusted  P Value^b^ | Controls with protein-truncating variants, No. (n=8846) | OR  (95% CI) | P Value^a^ | Adjusted  P Value^b^ |
| **Genes Significantly Associated With PDAC** | | | | | | | | | |
| **PALB2** | 7 (1.40%) | 15 (0.14%) | 10.03 (4.07-24.71) | 5.409E-07 | <0.001 | 7 (0.08%) | 17.97 (6.28-51.42) | 7.300E-08 | <0.001 |
| **BRCA2** | 13 (2.61%) | 34 (0.32%) | 8.30 (4.35-15.84) | 1.308E-10 | <0.001 | 20 (0.23%) | 11.80 (5.84-23.87) | 6.408E-12 | <0.001 |
| **ATM** | 5 (1.00%) | 19 (0.18%) | 5.63 (2.09-15.14) | 0.000617 | 0.018 | 28 (0.32%) | 3.19 (1.23-8.29) | 0.017 | 0.493 |
| **Genes NOT Significantly Associated With PDAC** | | | | | | | | | |
| **ERCC5** | 2 (0.40%) | 0 (0%) | 6.50E+6 (0-Inf) | 0.968 | >0.99 | 14 (0.16%) | 2.54 (0.58-11.20) | 0.219 | >0.99 |
| **CDH1** | 1 (0.20%) | 0 (0%) | 3.24E+6 (0-Inf) | 0.969 | >0.99 | 0 (0%) | 3.24E+6 (0-Inf) | 0.972 | >0.99 |
| **APC** | 1 (0.20%) | 1 (0.01%) | 21.26 (1.33-340.37) | 0.031 | 0.961 | 0 (0%) | 3.24E+6 (0-Inf) | 0.972 | >0.99 |
| **MSH6** | 2 (0.40%) | 5 (0.05%) | 8.52 (1.65-44.01) | 0.011 | 0.341 | 4 (0.05%) | 8.90 (1.63-48.68) | 0.011 | 0.319 |
| **RECQL4** | 4 (0.80%) | 18 (0.17%) | 4.75 (1.60-14.07) | 0.004996 | 0.155 | 24 (0.27%) | 2.97 (1.03-8.59) | 0.045 | >0.99 |
| **FANCM** | 2 (0.40%) | 10 (0.09%) | 4.26 (0.93-19.48) | 0.062 | >0.99 | 4 (0.05%) | 8.90 (1.63-48.68) | 0.011 | 0.319 |
| **BRCA1** | 3 (0.60%) | 16 (0.15%) | 4.00 (1.16-13.76) | 0.028 | 0.868 | 15 (0.17%) | 3.56 (1.03-12.39) | 0.045 | 1.395 |
| **FANCD2** | 2 (0.40%) | 14 (0.13%) | 3.04 (0.69-13.41) | 0.142 | >0.99 | 10 (0.11%) | 3.56 (0.78-16.27) | 0.102 | >0.99 |
| **NBN** | 1 (0.20%) | 7 (0.07%) | 3.04 (0.37-24.72) | 0.299 | >0.99 | 6 (0.07%) | 2.96 (0.36-24.62) | 0.316 | >0.99 |
| **ERCC2** | 1 (0.20%) | 7 (0.07%) | 3.04 (0.37-24.72) | 0.299 | >0.99 | 8 (0.09%) | 2.22 (0.28-17.77) | 0.453 | >0.99 |
| **RAD51C** | 1 (0.20%) | 8 (0.08%) | 2.66 (0.33-21.27) | 0.358 | >0.99 | 15 (0.17%) | 1.18 (0.16-8.97) | 0.871 | >0.99 |
| **SBDS** | 4 (0.80%) | 33 (0.31%) | 2.59 (0.91-7.32) | 0.074 | >0.99 | 91 (1.03%) | 0.78 (0.28-2.13) | 0.624 | >0.99 |
| **SDHA** | 1 (0.20%) | 9 (0.09%) | 2.36 (0.30-18.67) | 0.416 | >0.99 | 1 (0.01%) | 17.76 (1.11-284.37) | 0.042 | >0.99 |
| **FANCE** | 1 (0.20%) | 1 (0.20%) | 2.12 (0.27-16.63) | 0.473 | >0.99 | 1 (0.01%) | 17.76 (1.11-284.37) | 0.042 | >0.99 |
| **CHEK2** | 1 (0.20%) | 12 (0.11%) | 1.77 (0.23-13.64) | 0.584 | >0.99 | 21 (0.24%) | 0.84 (0.11-6.29) | 0.868 | >0.99 |
| **MRE11A** | 1 (0.20%) | 12 (0.11%) | 1.77 (0.23-13.64) | 0.584 | >0.99 | 9 (0.10%) | 1.97 (0.25-15.59) | 0.520 | >0.99 |
| **PMS2** | 1 (0.20%) | 13 (0.12%) | 1.63 (0.21-12.51) | 0.637 | >0.99 | 4 (0.05%) | 4.44 (0.50-39.79) | 0.183 | >0.99 |
| **ERCC4** | 1 (0.20%) | 16 (0.15%) | 1.33 (0.18-10.03) | 0.784 | >0.99 | 10 (0.11%) | 1.77 (0.23-13.89) | 0.585 | >0.99 |
| **BRIP1** | 1 (0.20%) | 20 (0.19%) | 1.06 (0.14-7.92) | 0.954 | >0.99 | 22 (0.25%) | 0.81 (0.11-5.99) | 0.833 | >0.99 |
| **FANCA** | 1 (0.20%) | 21 (0.20%) | 1.01 (0.14-7.53) | 0.992 | >0.99 | 29 (0.33%) | 0.61 (0.08-4.49) | 0.628 | >0.99 |
| **MSH3** | 1 (0.20%) | 22 (0.21%) | 0.96 (0.13-7.17) | 0.972 | >0.99 | 25 (0.28%) | 0.71 (0.10-5.24) | 0.736 | >0.99 |
| **BLM** | 1 (0.20%) | 23 (0.22%) | 0.92 (0.12-6.84) | 0.937 | >0.99 | 15 (0.17%) | 1.18 (0.16-8.97) | 0.871 | >0.99 |
| **WRN** | 1 (0.20%) | 27 (0.26%) | 0.79 (0.11-5.79) | 0.813 | >0.99 | 34 (0.38%) | 0.52 (0.07-3.81) | 0.520 | >0.99 |
| **MUTYH** | 1 (0.20%) | 27 (0.26%) | 0.79 (0.11-5.79) | 0.813 | >0.99 | 21 (0.24%) | 0.84 (0.11-6.29) | 0.868 | >0.99 |
| **RAD51D** | 1 (0.20%) | 28 (0.26%) | 0.76 (0.10-5.58) | 0.785 | >0.99 | 20 (0.23%) | 0.89 (0.12-6.62) | 0.906 | >0.99 |
| **PDE11A** | 1 (0.20%) | 121 (1.14%) | 0.17 (0.02-1.25) | 0.082 | >0.99 | 140 (1.58%) | 0.13 (0.02-0.90) | 0.038 | >0.99 |

Abbreviation: ChinaMAP, China Metabolic Analytics Project; gnomAD, Genome Aggregation Database; OR, odds ratio; CI, confidence interval.

^a^ Logistic regression analysis to establish P value.

^b^ Adjusted by Bonferroni correction for 29 genes.
